# Marginal Fit of Porcelain Laminate Veneer Materials under Thermocycling Condition: An In-Vitro Study

**DOI:** 10.3390/dj11010012

**Published:** 2023-01-01

**Authors:** Zanbaq Azeez Hanoon, Huda Abbas Abdullah, Zahraa Abdulaali Al-Ibraheemi, Rasha A. Alamoush, Suha Mohammad Sami, Julfikar Haider

**Affiliations:** 1 Department of Conservative Dentistry, Faculty of Dentistry, University of Kufa, Najaf 54001, Iraq; 2 Department of Conservative Dentistry, College of Dentistry, Tikrit University, Tikrit 34001, Iraq; 3 Prosthodontic Department, School of Dentistry, University of Jordan, Amman 11942, Jordan; 4 Maxillofacial Surgery Department, College of Dentistry, University of Kufa, Najaf 54001, Iraq; 5 Department of Engineering, Manchester Metropolitan University, Manchester M1 5GD, UK

**Keywords:** CAD/CAM, cervical marginal gap, laminate veneers, IPS e.max CAD, CEREC blocs, cervical marginal fit, thermocycling

## Abstract

*Objective*: The purpose of this study was to evaluate the cervical marginal fit of porcelain laminate veneer (PLV) restorations made from two different types of CAD/CAM ceramic laminates: CEREC C PC and E.max (LD). *Materials and Methods*: This in-vitro experiment used a total of 32 human maxillary first premolars that were clean and free of any cracks or caries, extracted for orthodontic purposes. The samples were divided in a random way into two study groups: A and B (*n* = 16). Each sample was mounted on a dental surveyor and a silicon impression was made to create a silicone index for each tooth in both groups. Standardized preparation was carried out for all the samples by using preparation bur kit for the ceramic veneer system. Subsequently, digital impressions were made for all the samples by using Trios 3 shape intraoral camera (Sirona Dental Systems). The design of veneer restorations was made using Sirona inLab CAD SW 16.1 with CEREC inLab MC XL (Dentsply, Sirona Dental Systems, Bensheim, Germany). The veneer restorations were cemented using 3M RelyX veneer resin cement (3M ESPE, Seefeld, Germany) and the samples kept in distilled water for two weeks at 37 °C. All the specimens were subjected to thermocycling in a water bath with temperature varying between 5 °C and 55 °C for 500 cycles. The cervical marginal fit of veneers was evaluated by a digital microscope after sectioning the embedded teeth in acrylic resin. *Results*: The lowest mean of cervical marginal gap was recorded for Group A (91.59431 ± 1.626069) which was restored with CEREC CAD/CAM veneers, while the highest mean value of the gap was recorded for Group B (106.48863 ± 2.506684) which was restored with IPS E.max CAD. The *t*-test showed that the type of porcelain veneer restoration had a highly significant effect on the cervical marginal fit (*p* ≤ 0.01). *Conclusions*: CEREC CAD/CAM veneers showed smaller cervical marginal gaps, indicating a better fit compared to the IPS E.max CAD.

## 1. Introduction

Porcelain laminate veneer (PLV) restorations have become very popular options for the aesthetic enhancement of the anterior teeth, especially those with discoloration, fractures and malformations [1]. Furthermore, PLV can be used to aesthetically improve the shape of anterior teeth and to eliminate large spaces between them, known as diastema closure, which requires full coverage restoration. IPS e.max CAD ceramic was known as lithium-disilicate (LD) ceramic material that can be used with computer-aided design and computer-assisted manufacturing (CAD/CAM) technology. IPS e.max CAD ceramic shows a great spectrum of features, such as excellent translucency in comparison with other ceramic core materials. CEREC Blocs C PC is another glass ceramic material, namely feldspathic ceramics with delicate structure that are constructed as a block to be used with CAD/CAM technology, and was developed with excellent optical characteristics [2,3,4].

One of the most important factors affecting the long-term success of restorations is the marginal fit to the prepared areas. The marginal discrepancy vertically and horizontally both have a strong relation with the fit of the restoration margin. The vertical interspace that located between the inner surface of the restoration and the finishing point of the preparation is known as the marginal gap. The sealing of these gaps that oriented vertically at the margin of the restorations can only be done using luting cement, which is characterized by roughness, porosity, and subject to dissolution. The bigger the marginal gap, the faster that luting cement can be dissolved [5,6,7,8].

The fabrications of indirect restoration will always involve a marginal gap, and the presence of the marginal gap contributes to continuous cement degradation, secondary caries and periodontal problems. For that reason, clinicians look to minimize the marginal gaps, which in turn lead to a reduction in the rate of teeth staining, inflammation of the gingiva, dental caries and periodontal problems associated with the surface roughness that appears due to the luting cement degradation. Clinically, there is no evidence-informed consent concerning whether a particular marginal gap may be clinically suitable for a specific patient. A few studies showed that a marginal fit of ≤100 µm is generally acceptable [9,10].

Numerous factors contributed to the precision marginal fit including factors associated with tooth preparation where the margin is placed supragingivally or subgingivally, features of the milling machine, milling burs, type of digital scanner system and so on. During construction of the veneer restorations utilizing CAD/CAM technology, prolonged success rate depends on the type of digital scanner system used in the scanning of the prepared tooth surface, which in turn affects the marginal fit of the restoration [11,12,13].

The usage of an intraoral scanner for the impression of the prepared area is preferred over the usage of the conventional method of impression making, due to a number of advantages such as easier workflow, exorbitant precision scanning of the recommended area, advanced software, and standardized milling procedure. That highly contributes to the reduction of the marginal inconsistency of the restoration made by CAD/CAM technology. Applying digital technologies aid in more precise restoration fit due to magnification of the scanned area [14,15].

After extensive literature review, it was found that no study reported on the performance of CEREC C PC PLV in terms of marginal fit. Therefore, it is important to evaluate the performance of the CEREC C PC PLV, which ensures its suitability in clinical applications. The aim of this work is to compare the marginal fit between two CAD/CAM veneer ceramics: CEREC C PC and IPS e.max CAD. The hypothesis assumed here is that there will be no significant differences in the marginal fit between the two veneer materials considered in this study.

## 2. Materials and Methods

### 2.1. Tooth Collection and Grouping

A total of 32 maxillary first premolars free from caries and cracks were used in this study after extracting them for orthodontic purposes. Further inclusion criteria include vital tooth without any root canal or other types of filling, similar size and shape, double root teeth, and patients aged between 16 and 35 years. These teeth were subjected to visual examination by the blue light transillumination to make sure the crown of each tooth was free of any cracks. In order to confirm the similarity in the dimensions of the teeth, the buccopalatal, mesiodistal and occlusogingival dimensions were measured using a digital caliper with an accuracy of 0.01 mm. The mean and standard deviation values of buccopalatal (8.48 ± 0.176 mm), mesiodistal (7.39 ± 0.175 mm) and occlusogingival (8.55 ± 0.169 mm) dimensions showed good homogeneity. The standard deviation values were much less than 10% of the corresponding mean values of the measured dimensions. After cleaning and removing the residue of attached soft tissue, hand scaling and polishing with pumice paste free from fluoride (Produits Dentaires S.A, Vevey, Switzerland) was performed on the teeth. Saline was used as the solution for storing the teeth at room temperature up to the time of the experiment [16]. Consent was obtained from the patients that the extracted teeth will be used for research purposes.

Teeth were divided in a random way into two groups (*n* = 16) as follows:

Group A: Teeth were restored with veneers constructed from CEREC CAD/CAM blocks (CEREC C PC, Dentsply Sirona, Bensheim, Germany).

Group B: Teeth were restored with veneers constructed from lithium disilicate ceramic CAD/CAM blocks (IPS e.max CAD, Ivoclar/Vivadent, Germany).

A researcher who was not involved with the tooth collection and measurement randomly divided the teeth into two groups to avoid any bias. Afterwards, each group was divided into two subgroups randomly and one way ANOVA did not show any significant differences within a group or between the groups when the buccopalatal, mesiodistal and occlusogingival dimensions data were used.

### 2.2. Veneer Preparation Procedure

**Figure 1:**
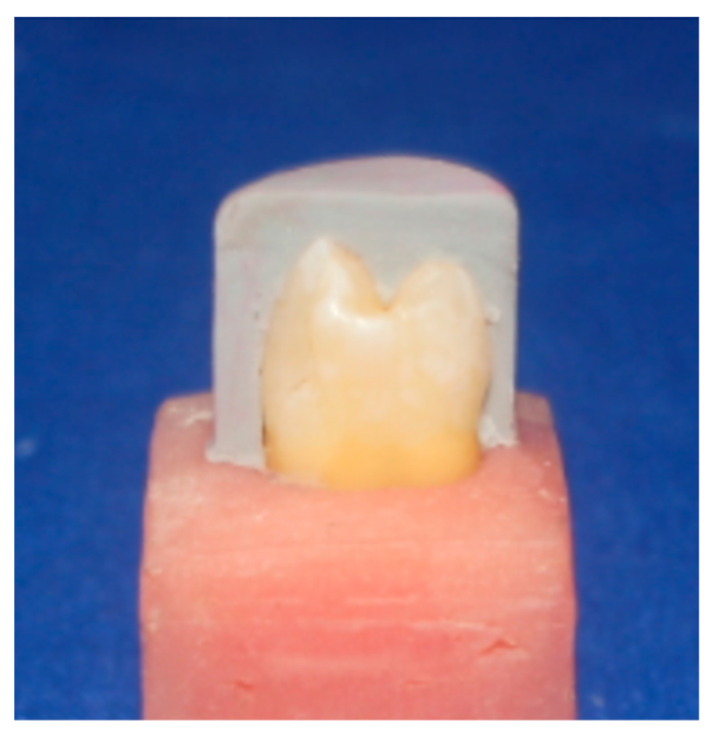
Silicone index made from condensation silicone.

**Figure 2:**
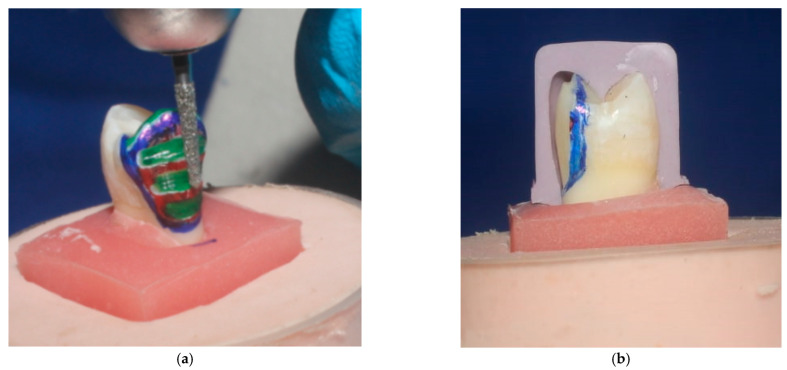
(**a**) Veneer preparation with tapered fissure diamond bur used for the removal of the tooth structure between the depth grooves and (**b**) Silicone index to check the preparation.

Dental surveyor (Paraline, Dentaurum, Ispringen, Germany) was used for mounting of the tooth samples. Silicone index was fabricated from putty condensation silicon impression material (Protesil, Italy) for every tooth in the two groups prior to the preparation of the teeth (Figure 1), so that the precision of tooth reduction could be carefully assessed [17]. Standardized preparation was performed for each tooth by utilizing preparation bur kit (keramik-Veneer. De, Komet, Germany) for the ceramic veneers system (Figure 2). The reduction of the buccal aspect was carried out by grinding 0.4 mm cervically, and 0.5 mm occlusally at the middle. After the finishing of the preparation, impression was performed digitally by the help of an intraoral camera known as Trios 3 shape (Sirona Dental Systems, Copenhagen, Denmark).

Sirona inLab CAD SW 16.1 was utilized with CEREC inLab MC XL (Dentsply, Sirona Dental Systems, Bensheim, Germany) for the designing of the veneer restorations. The veneers made from CEREC C PC blocks (Group A) were in fully crystallized condition after milling, but they require further characteristics, which were made with VITA accent paints (VITA, Germany) in a firing furnace (Programat p500, Ivoclar Vivadent /technical, Liechtenstein, Germany). On the other hand, veneers made from IPS e.max CAD blocks (Group B) had a violet bluish color, which indicated that they were in pre-crystallized condition after milling, so they were subjected to crystallization in a furnace in order to match to the shade of the tooth [4].

### 2.3. Cementation of Veneer

Etching internal surface of the veneers was carried out for 90 s with 9% hydrofluoric acid gel (Ultradent Porcelain Etch, Ultradent Products, South Jordan, UT, USA) in accordance with the manufacturer’s instructions, followed by rinsing and air drying. Phosphoric acid etching gel (37%, SDI super Etch, SDI, Australia) was applied to the inner surface of the veneers to remove the byproducts that precipitated on the inner surface of the restoration, and washing and air drying of the veneers was then completed. The inner surface of veneer restorations was covered with silane coupling agent (Ultradent Porcelain Etch, Ultradent Products, South Jordan, UT, USA) in order to enhance its bonding with the luting cement.

**Figure 3:**
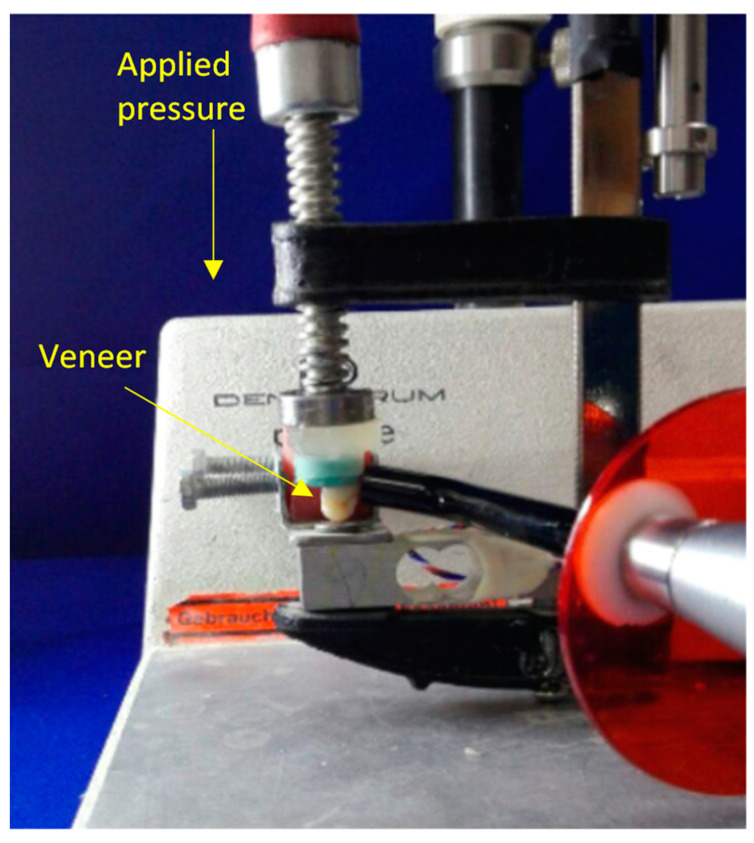
Custom made cementation device.

After cleaning and etching of all the prepared teeth, Single Bond Universal Adhesive (3M ESPE, Seefeld, USA) was applied to the prepared teeth and to the silanized surface of the veneers. The cementation was made with 3M RelyX veneer resin cement (3M ESPE, Seefeld, Germany). After the application of the resin cement on the internal surface of the veneers, these veneers were seated in occluso-cervical direction on the prepared teeth with the help of Optrastick (Ivoclar Vivadent/clinical, Germany) under light pressure. The positioning pressure was controlled by a custom-made cementation device at 1 kg [18,19] (Figure 3).

Light curing (Perfection plus, Totton, UK) of each veneer was performed for about forty seconds buccally, mesially, distally and occlusally. Finally, finishing and polishing of the margins was conducted (Polishing Disc DENCO PACK /40, Germany). The finished samples were stored in distilled water for two weeks at 37 °C.

### 2.4. Thermocycling Procedure

In an effort to create simulation to the environment of the oral cavity, all samples were submitted to thermal cycles. An automatic thermocycling device was used to perform the procedure with 500 cycles in water by cycling them between two water containers where the temperature of the first container was kept at 5 ± 0.5 °C and the other one at 55 ± 0.5 °C, with a dwell time of at least 20 s [3] based on the recommendations made by International Organization for Standardization (ISO TR 11405).

### 2.5. Marginal Fit Evaluation Procedure

**Figure 4:**
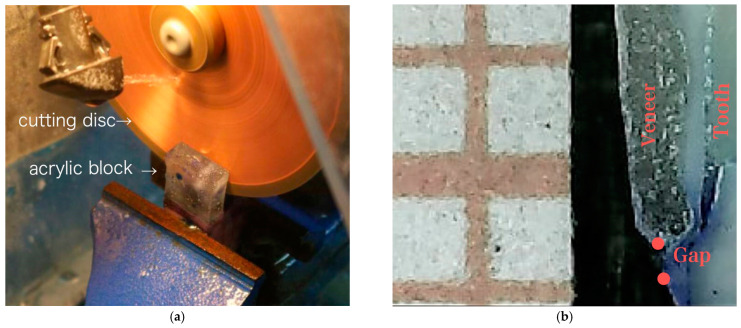
(**a**) Sectioning using Microtome cutter (**b**) Vertical section of veneer demonstrating marginal fit at the cervical region.

In this study, the evaluation of marginal fit was conducted by measuring the maximum distance between the finishing point of the underlying prepared tooth and the margin of the ceramic laminate veneer on the cervical margin directly through vertical sectioning procedure, which is an extremely advantageous tool that helps in reducing the chances of software and repositioning errors and permits an undisturbed view of the marginal gap (Figure 4). In order to reduce the chances of specimens’ destruction during the sectioning procedure, acrylic resin was used, into which the specimens were embedded [20,21,22,23,24].

During the sectioning procedure, a single position for the seating of the samples in the sectioning machine (Microtome, MT-4 Diamond cut-off saw, Cleveland, USA) was selected in an attempt to create a standardized cutting area through the samples. The thickness of the cutting blade used during the cutting procedure was 0.3 mm. A digital microscope was utilized to estimate the vertical space between veneer and tooth at a predetermined point in the cervical area. In order to view and measure the marginal gap precisely, a magnification ×230 was selected, and Image J software used to measure the gap. A predetermined measuring point at the cervical margin was chosen for each specimen and a mean of three readings was taken for each sample [4,25]. All experimentations were conducted at a room temperature of 25 °C and a relative humidity of 40% to avoid any risk of bias error. An experienced dentist carried out the marginal fit evaluation without being given any idea about the groupings or materials used.

### 2.6. Statistical Analysis

Statistical analysis was carried out by SPSS 2021 to check the distribution of data and the result of the test shows the data were normally distributed, and the differences in the data did not affect the overall result. Descriptive statistics were employed to compare the mean marginal gaps between the veneer materials. A *t*-test was used to detect whether there were significant effects of the type of porcelain veneer restoration on the cervical marginal fit.

## 3. Results

A total of 32 samples were measured and the cervical marginal gap was measured three times for each sample for the experimental groups. The minimum and maximum with the means and standard deviations of the cervical marginal gap values were calculated and shown in Table 1.

The lowest mean of the cervical marginal gap was recorded for Group A (91.59431 μm) which was restored with CEREC CAD/CAM, while the highest mean value of the gap was recorded for Group B (106.48863 μm) which was restored with IPS E.max CAD veneers. The result of the *t*-test (Table 2) showed the type of porcelain veneer restoration had a highly significant effect on the marginal fit (*p* ≤ 0.01).

## 4. Discussion

PLVs are considered to be a suitable solution for dental procedures that require changes in the arrangement of the anterior teeth, their size, shape or color. Laminate veneers are also considered as conservative options for the management of teeth with congenital malformation, discoloration or fracture, and for reshaping of the anterior teeth or closing of multiple diastemas instead of full coverage restorations with minimal preparation. Therefore, two different PLV materials were used to evaluate the marginal fit [26]. The precise marginal adaptation of indirect restoration is associated with a minimal marginal gap between the indirect restoration and the prepared tooth. The significance of the marginal fit arises from the fact that the main causes for lack of success of indirect restorations are recurrence of caries and cessation of luting resin cement, which can result in poor retention of the restoration [27,28]. The hypothesis was rejected as the result of the current study showed that the type of porcelain veneer restoration had a highly significant effect on the marginal fit (*p* ≤ 0.01).

The accuracy of tooth reduction can be controlled by different clinical methods including silicone index and depth limiting burs [29,30]. In this in-vitro study, all teeth received a standardized buccal reduction utilizing the diamond depth cutter burs (Ceramic Veneer Set, Komet, Germany) to ensure equal reduction of approximately 0.4 mm depth cervically was obtained, as the thickness of the enamel in the cervical area of the teeth does not allow preparation of 0.5 mm without exposing the dentin, and 0.5 mm on the occlusal two thirds. Such reduction depths were made to ensure that the whole preparation was within the enamel [31,32,33]. At the occlusal surface, cuspal reduction was conducted to maintain a low stress concentration in comparison to the occlusal preparation without cuspal reduction, in which the buccal cusp reduction was 1.5 mm occlusio-cervically and 1.5 mm from the tip of the cusp palatally placing the veneer margins out of the contact and grooves occlusally [34,35].

In this study, the inner surfaces of porcelain veneers were subjected to etching with hydrofluoric acid, as well as salinization, and they were bonded to the prepared teeth which had been manipulated utilizing an etch and rinse adhesive according to the standard bonding procedure. It was confirmed by numerous studies that the best enamel sealing of the cemented veneers was achieved by the etch and rinse resin luting cements [36,37].

In this study, RelyX veneer cement (3M ESPE, Seefeld, Germany) was used as a light-cure resin cement for the cementation of the veneers to the preparations. Because veneer restorations are delicately thin and transparent that permit the curing light to pass through it which is necessary for the complete polymerization of the cement, a light-cured resin cement was utilized [38]. The RelyX veneer cement was chosen because of its capability to be effectively light-cured when exposed to visible blue light in the range of 400–500 nm. Another feature of the RelyX veneer cement is the absence of unreacted amines that are necessary for the integration with the peroxides in the catalyst of dual-cured cement, to which is attributed its remarkable color stability. In addition, the RelyX veneer cement is characterized by the presence of dimethacrylate polymer which adjusts the physical features of the material that result in excellent flowability under the applied pressure but which, at the same time, keeps its shape and remains in place until it is light-cured (3M ESPE, 2010).

Thermocycling procedure was highly advised to be applied in the in-vitro studies in order to mimic the environment of the oral cavity. The different ranges of the temperature in the oral environment contributed to stress formations in the restoration and can also result in a negative effect on the sealing integrity of the margins. The sealing potentiality of the luting resin cement and its resistance to the different stresses are also very important elements that impact the extent of existing gap and marginal leakage. The prolonged survival rate of the porcelain veneer restorations in the oral cavity depends on the strength and firmness of the bond between tooth surface over the time, the resin cement, and the porcelain veneer restoration. The exposure of resin luting cement to the fluids of the oral environment can result in various issues such as water sorption, polymerization shrinkage, wear, microleakage and large marginal discrepancies which occurred due to vulnerability of the luting material [39,40,41]. It was reported that thermocycling procedure would lead to shrinkage of the samples due to exposure to cold water followed by expansion in hot water which, as a consequence, would increase the marginal gap. Consequently, the differences in the coefficients of thermal expansion between the restorations, the luting cement and the dental tissue lead to generation of stresses on the bonding interface. These stresses are anticipated to enlarge the width of the existing gaps or develop new gaps. The differences between expansion and contraction probably promoted an increase in the marginal gap [42,43,44]. The thermocycling procedure on the samples in this study contributed to the generation of remarkable stresses in the veneers [45].

Measurement of the marginal gap at the cervical region was performed by measuring the vertical distance between the veneer and tooth at the predetermined points in the cervical area in μm (Figure 4b), in which the measurement points were chosen for each specimen and a mean of three readings was taken for each sample. All the measurements were completed using an image processing software. The precision of this method in measuring is more preferable due to its reduced opportunity for generating bias compared to other methods. Maximum clinically acceptable marginal gap distance values have been reported to be between 100–150 µm [46,47]. In this study, for both the materials, the marginal gaps were either close to or less than the lower range. This indicated that both materials are suitable as good PLV with smaller gaps, which could help in long-term clinical success.

The best method to measure the marginal gap still remains a debatable topic. Although the most common procedure is sectioning of the restorations and measuring the discrepancies under a light or a Scanning Electron Microscope (SEM), more recently, micro-computed tomography (micro-CT) allows for a non-destructive evaluation of the marginal gap [48]. However, the disadvantage of the technique includes low capacity for discrimination of CT micro-tomography when compared with an optical or electron microscope (1.8 μm for micro-tomography and 0.3 μm and 0.25 nm for optical and electron microscopes, respectively). In addition, considering that the images result from radiation, there might be artifacts from refraction. The more materials with different coefficients of absorption that exist, the more difficult it is to clearly define the lines between those materials. This has to be considered when planning to use a micro-CT for marginal gap evaluation [49].

The veneers in this study were made from two different CAD/CAM ceramic materials: IPS e-max CAD which is lithium disilicate glass ceramic, and CEREC C PC which is feldspathic ceramic. Each material is characterized by unique features such as the excellent aesthetic and strength (360 Mpa) (Ivoclar Vivadent, 2010), while the latter shows excellent integration within the tooth structure and outstanding natural appearance of the veneer restoration (Dentsply Sirona, Bensheim, Germany). CEREC C PC is a feldspathic fine-grained ceramic fabricated to obtain a flawless material which could be machined using the CEREC system (Dentsply Sirona). The development of this material enabled the incorporation of different shades and translucencies of dentine in the same block to mimic the polychromatic nature of the tooth [50]. On the other hand, lithium disilicate glass ceramic is the most favorable material utilized for all ceramic restoration. The lithium disilicate is an amorphous glass matrix that was converted to a crystalline material with about 70% of lithium disilicate orthorhombic crystal phase when subjected to heat treatment. The translucency and exquisite aesthetic appearance of these glass ceramics materials made them a preferable option compared to the polycrystalline alternative materials [51,52,53].

According to the results of the *t*-test, a highly significant effect was found for the type of ceramic restoration on the marginal fit of the porcelain veneers. This finding was in agreement with Aboushelib et al. [22], who concluded that pressable ceramic laminate veneers produced a higher marginal adaptation and smaller marginal gap formation compared to the machinable ceramic veneers. Lin et al. reported that the type of ceramic restoration had a significant effect on the marginal gap formation and marginal fit of the porcelain veneers that were restored with leucite-reinforced ceramic (ProCAD) and Noritake Super Porcelain EX3 [54]. However, these observations disagreed with the findings of Hekimoglu et al. [55], who concluded that the type of porcelain material had no significant effect on the marginal fit. Soares-Rusu et al. [56] found similar disagreement with no statistical differences in the marginal adaptation between CAD/CAM (IPS e.max CAD) and heat-press (IPS e.max Press) veneers. This may be caused by the usage of different ceramic materials, different restoration methods, use of thermocycling procedure and different methods of marginal gap measurement. The *t*-test showed highly significant difference between the groups (A and B), and C PC showed better marginal fit than IPS e.max CAD. This finding agreed with the results obtained by Basheer et al. [57], who claimed that the highest statistically significant marginal gap distance was recorded with Prettau followed by IPS e.max CAD, while the lowest statistically significant marginal gap was obtained for VITA SUPRINITY. It should be noted that no previous studies compared the marginal fit of both the ceramic materials used in this study.

Micro-gap formation at the weaker bond interface cervically can result from contraction stress that was created by polymerization shrinkage of the resin cement. The differences in coefficient of thermal expansion between the different types of porcelain restoration materials and the tooth can also create stress at the bond interface which occurs during the temperature change, resulting in micro-crack propagation and eventually gap formation and microleakage [24,58,59]. Larger gap formation was found at the tooth/luting resin cement interface, as compared to the porcelain/luting resin cement interface. The micro-topical irregularities found in the etched enamel or dentin surface were less prominent as compared to that in the etched porcelain surface, which results in an excellent adhesive strength between the etched silanized porcelain-luting resin (33 MPa), and it was significantly higher than the luting resin-etched enamel bond strength (31 MPa). Polymerization shrinkage of the resin cement led to stress formation at the weaker bond interface and subsequent micro-gap propagation [59,60,61].

In order to create a better marginal fit, it is sensible to choose restorative materials with coefficient of thermal expansion (CTE) close to the CTE of the enamel (11.4 × 10^−6^ K^−1^) and dentin (8.3 × 10^−6^ K^−1^) of the natural tooth, which would lead to lesser gap formation between the porcelain/tooth interface due to less thermal stress developed in the oral cavity [24,62]. CEREC C PC ceramic has the smallest difference and closest coefficient of thermal expansion (9.5 × 10⁻⁶ K⁻¹) to both enamel and dentin, whereas the IPS e.max ceramic (10.6 × 10⁻⁶ K⁻¹) is close to CTE of the enamel but substantially different to CTE of the dentin. This might be the reason for the reported better marginal fit of CEREC C PC veneer that translated into a better result during the thermal changing of the material.

In order to reduce the chance of marginal gap formation, the use of a material with lower modulus of elasticity was also suggested. CEREC C PC had a significantly lower modulus of elasticity (45 ± 0.5 GPa) in comparison with the IPS e.max ceramic (95 ± 5 GPa). The combination of the lower modulus of elasticity and lower strength of CEREC C PC ceramic translated into higher resiliency compared to IPS e.max ceramic. This resulted in better elastic buffer and compensation for resin cement shrinkage stress, which could be another explanation for the better marginal fit of the CEREC C PC veneers [4,63].

Mechanical performance and eventual functional success of a restoration depend on porcelain veneer preparation design and geometry. Zarone et al. established guidelines for veneer preparation to restore the maxillary anterior teeth [64]. They recommended a chamfer preparation for central incisors, a window preparation for canines, or both preparations for lateral incisors. In another study, ceramic veneer with 1 mm incisal reduction and 1 mm height of palatal chamfer showed the highest fracture resistance as compared to 1 mm incisal reduction with butt joint and no incisal reduction with facial-incisal bevel [65]. The palatal chamfer margin results in preservation of some peripheral enamel layer, which eliminates the micro leakage at the palatal margin-restoration interface and effectively counteracts shear stress. This design provides a definite seat for cementation.

Sorrentino et al. evaluated the amount of dentin exposure for window (WI) and butt joint (BJ) designs of tooth preparation for laminate veneers. The assessment was carried out by 3 operators with different clinical expertise. For the BJ preparation, no difference was found in detecting the exposure of dentin by the operators. On the other hand, for the WI preparation, the general practitioner and the other two operators found differences in dentin exposure. It was also found that the amount of dentin exposed was not associated with the two tooth preparation designs considered [66].

The main limitation of this study was that restoration was carried out on the extracted teeth outside the oral cavity with an attempt to mimic the natural condition of the oral environment by using thermocycling procedure. Furthermore, single preparation design and a single fabrication technique of veneer were applied, instead of other fabrication techniques such as pressable versus machinable ceramic laminate veneers. Other techniques for measuring the marginal fit of restorations such as direct measurement, profilometry, micro-CT technique and silicone replica technique might produce slightly different results compared to this study. In addition, the marginal fit was evaluated at the cervical margin only, instead of both cervically and incisally.

## 5. Conclusions

Within the limitations of this in-vitro study, which include using extracted teeth instead of working on teeth inside the oral cavity, the use of different aging factors, different resin cement, different preparation design, and different impression techniques, it can be concluded that the type of dental ceramic restoration had highly significant effects on the cervical marginal fit of the porcelain veneers made from lithium disilicate (IPS e.max CAD) and feldspathic (CEREC C PC) ceramics. CEREC C PC veneers showed significantly better marginal fit in comparison with the IPS e.max CAD veneers.

## Figures and Tables

**Table 1:** Descriptive statistical results for the tested groups.

Group	Mean Marginal Gap (μm)	*n*	Std. Deviation (μm)	Minimum (μm)	Maximum (μm)
CEREC CAD/CAM	91.594	16	1.626	88.143	93.807
IPS e.max CAD	106.489	16	2.507	101.210	109.807

**Table 2:** Independent samples *t*-test.

		Levene’s Test for Equality of Variances		*t*-Test for Equality of Means						
		F	Sig.	t	df	Sig. (2-Tailed)	Mean Difference	Std. Error Difference	95% Confidence Interval	
									Lower	Upper
Marginal fit	Equal variances assumed	2.055	0.162	−19.939	30.000	0.000	−14.894	0.746	−16.419	−13.368
	Equal variances not assumed			−19.939	25.725	0.000	−14.894	0.746	−16.430	−13.358

## Data Availability

The data presented in this study are available within the article.
